# Supplementary material for: Assessment of differentially methylated loci in individuals with end-stage kidney disease attributed to diabetic kidney disease: an exploratory study
Source: Clin Epigenetics. 2021 May 1;13:99. doi: 10.1186/s13148-021-01081-x (PMC8088646; doi:10.1186/s13148-021-01081-x)
Supplement: Supplementary file 2 — Additional file 2. Supplementary Figures. [file 13148_2021_1081_MOESM2_ESM.docx]

**Supplementary Figures (SF)**


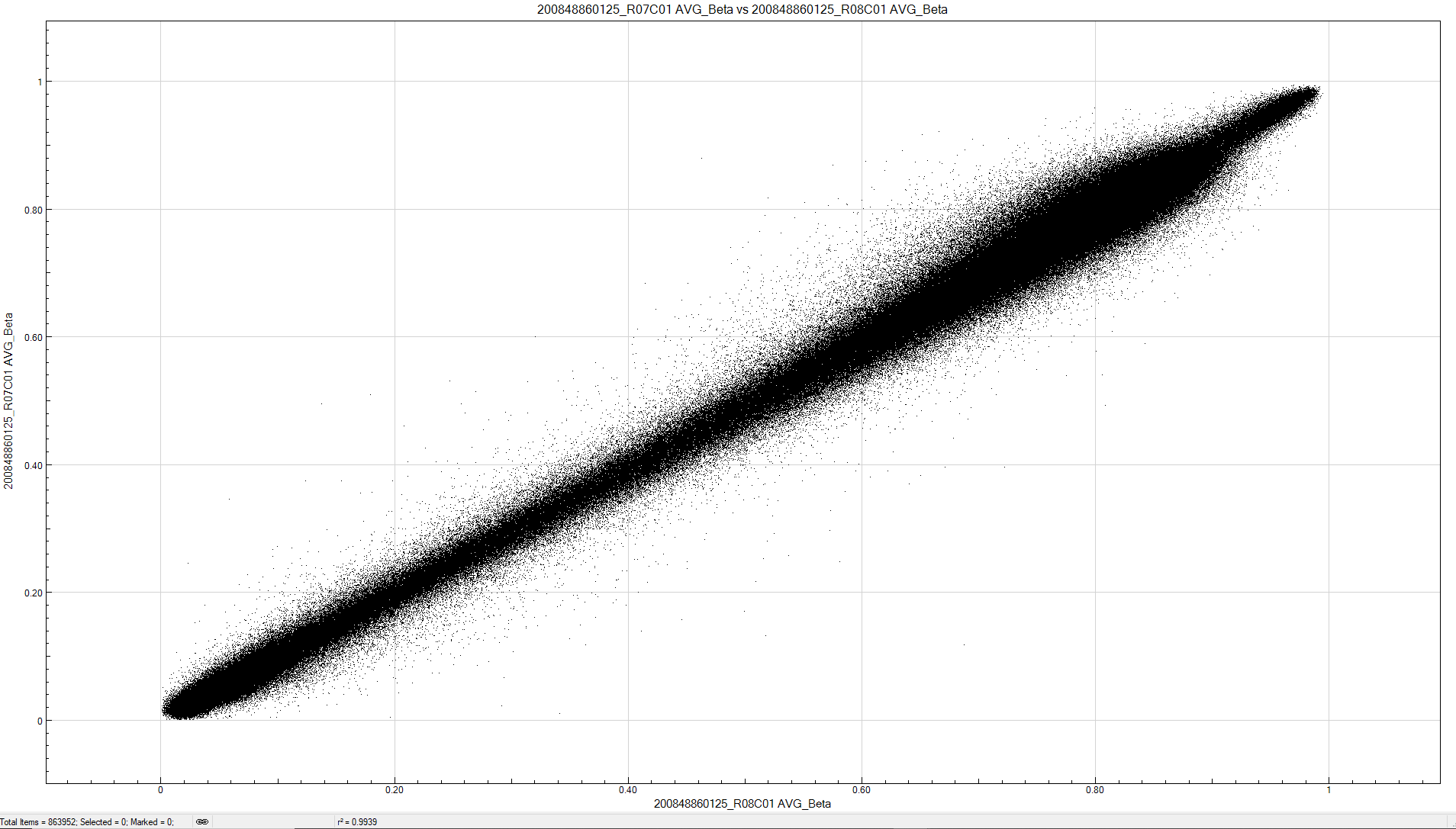


**SF1.** Representative concordance plots for a duplicate sample pair - average r^2^ for seven duplicates = 0.99.


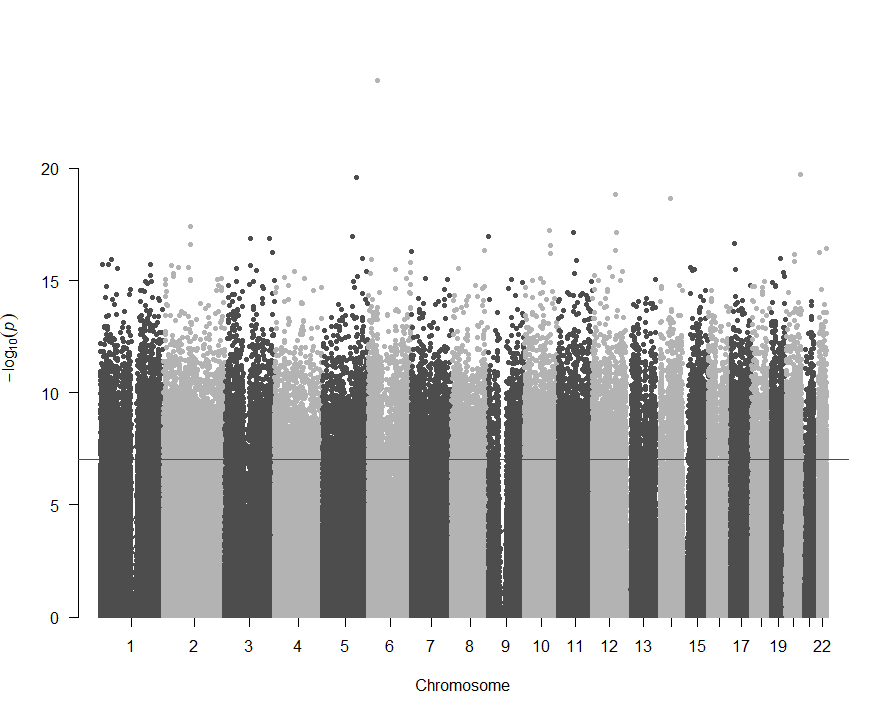


cg03546163 - *FKBP5*

cg12378834 - *C5orf66*

cg03754620

**SF2.** *Analysis 1* Manhattan Plot

**
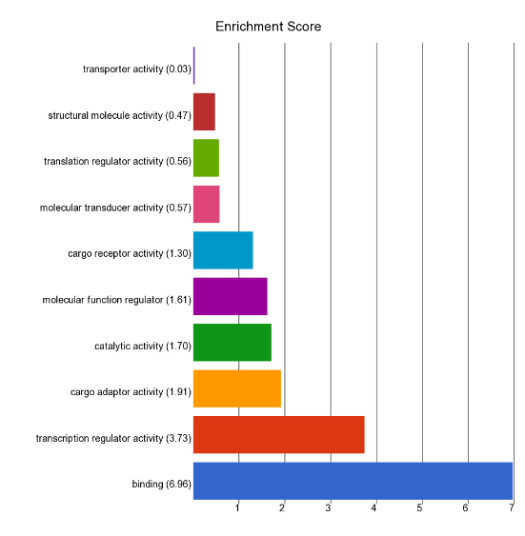

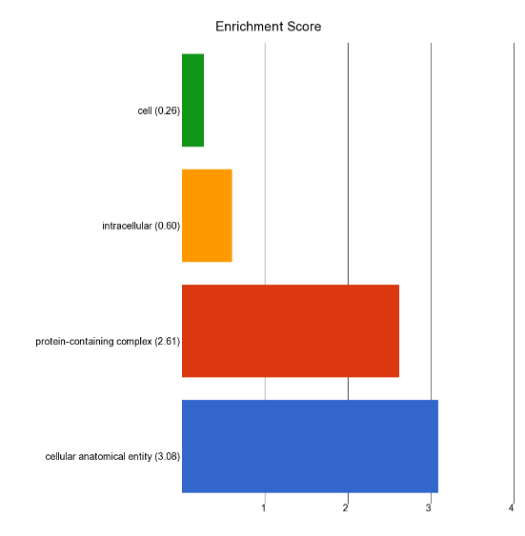
**

**
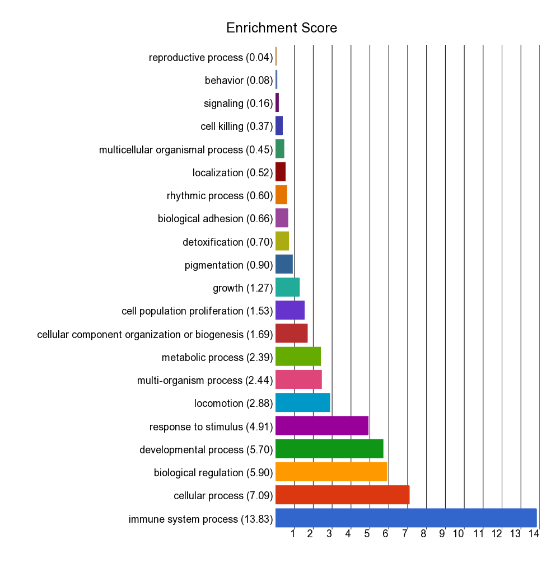
**

**SF 3a. GO analysis of biological processes SF 3b. GO analysis of cellular components SF 3c. GO analysis of molecular functions**

**SF3.** GO enrichment results for *Analysis 1*. Enrichment of top-ranked CpG sites for matched individuals with T1DM-ESKD (n=107) vs. T1DM (n=107): (FDR p-value ≤x10^-8^ and FC ±2). These gene classes are over-represented in the disease phenotype compared to a control gene set.

Abbreviations: CpG, cytosine-phosphate-guanine; ESKD, end-stage kidney disease; FC, fold change; FDR, false discovery rate; GO, gene ontology; T1DM, type 1 diabetes mellitus


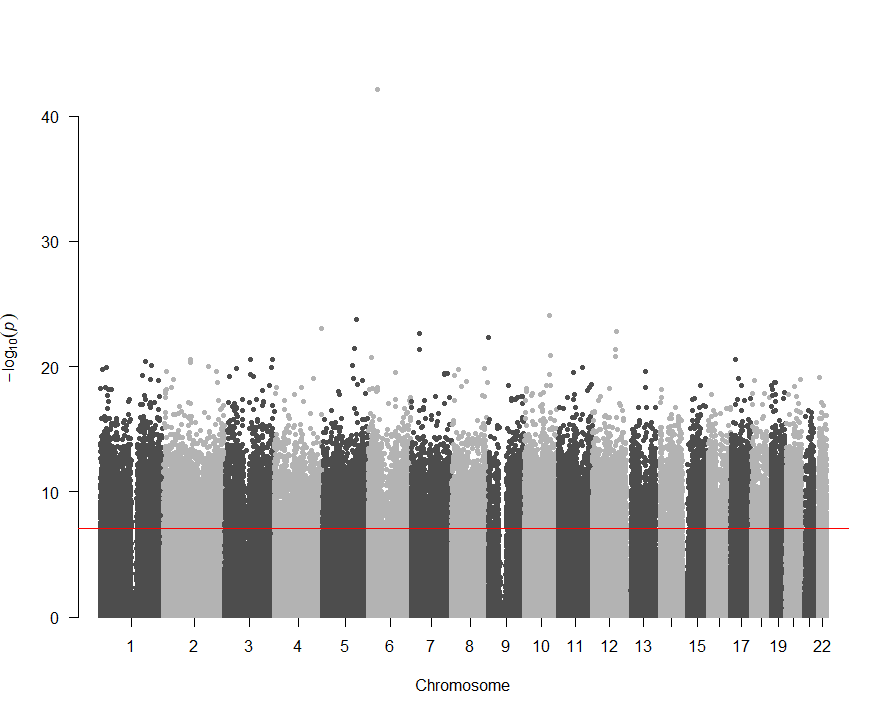


cg03546163 - *FKBP5*

cg24825894 - *PIK3AP1* - *C5orf66*

cg12378834 - *C5orf66*

**SF4.** *Analysis 2* Manhattan Plot


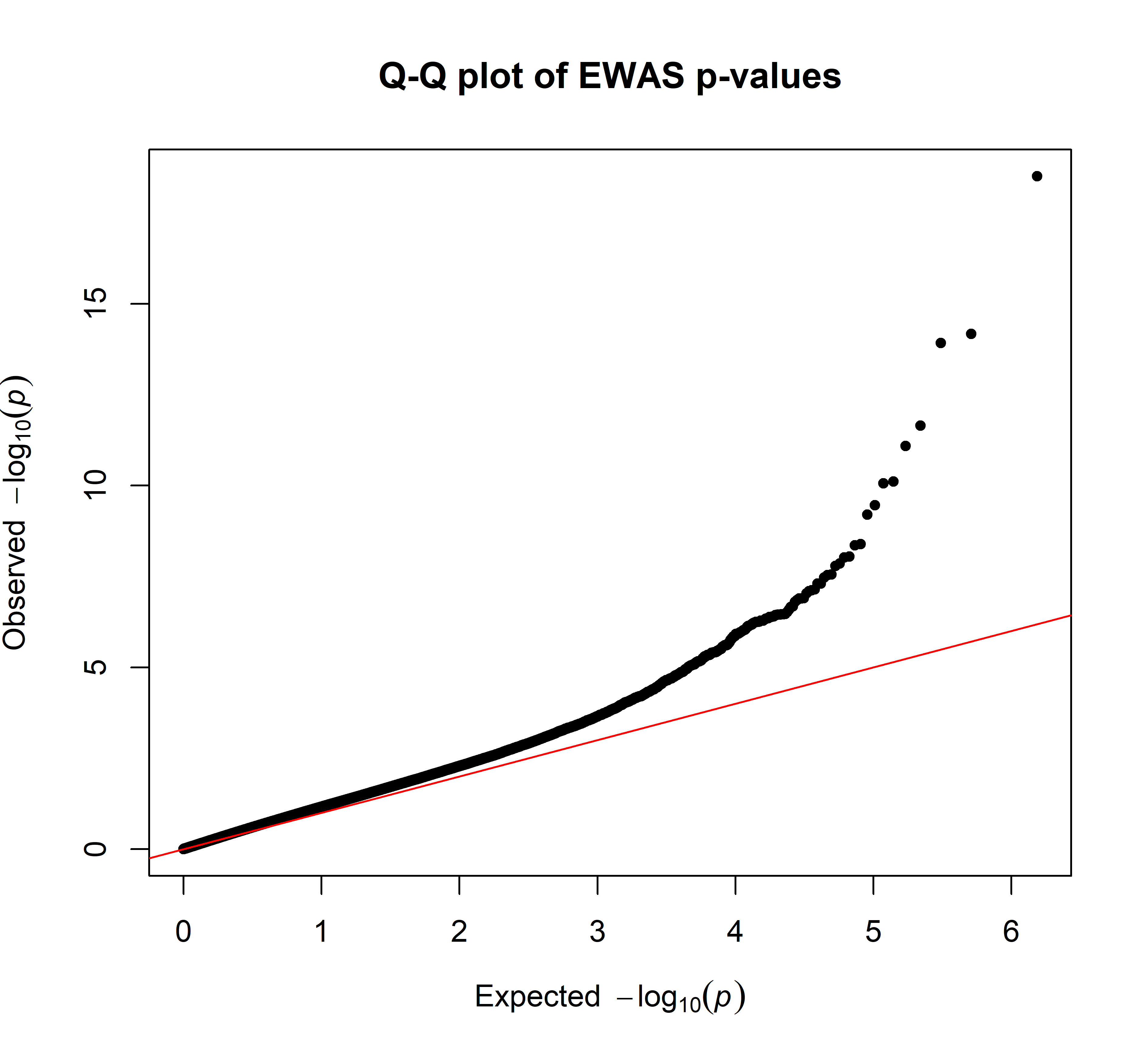


**SF5.** *Analyses 1 and* *2* QQ Plot

**
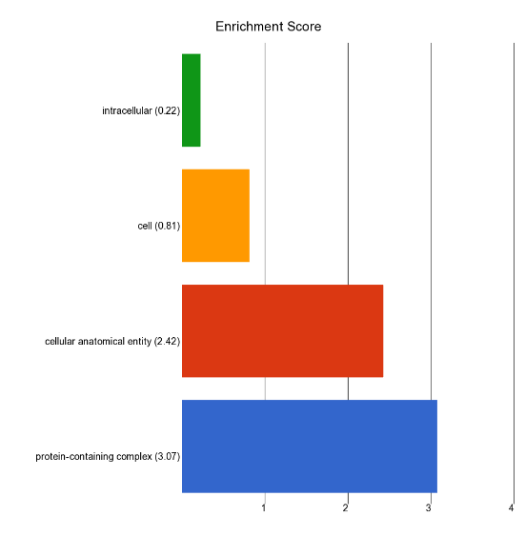

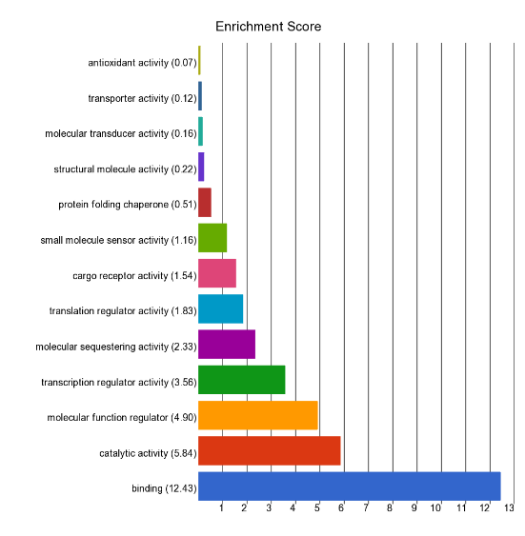

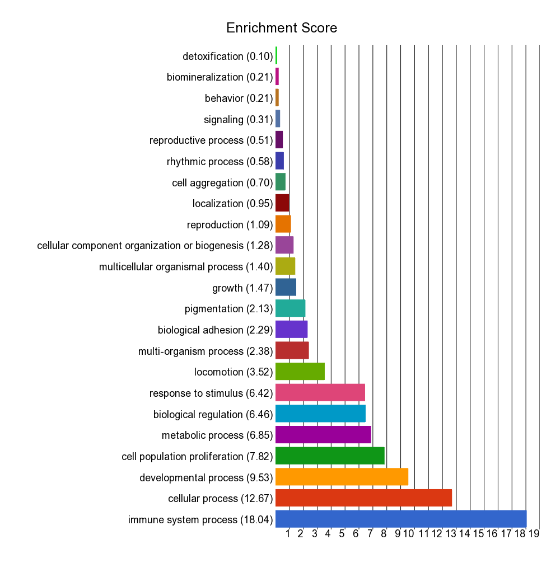
**

**SF 6a. GO analysis of biological processes SF6b. GO analysis of cellular components SF 6c. GO analysis of molecular functions**

**SF6.** GO enrichment results for *Analysis 2*. Enrichment of top-ranked CpG sites for matched individuals with T1DM-ESKD (n=107) vs. T1DM (n=253): (FDR p-value ≤x10^-8^ and FC ±2). These gene classes are over-represented in the disease phenotype compared to a control gene set.

Abbreviations: CpG, cytosine-phosphate-guanine; ESKD, end-stage kidney disease; FC, fold change; FDR, false discovery rate; GO, gene ontology; T1DM, type 1 diabetes mellitus


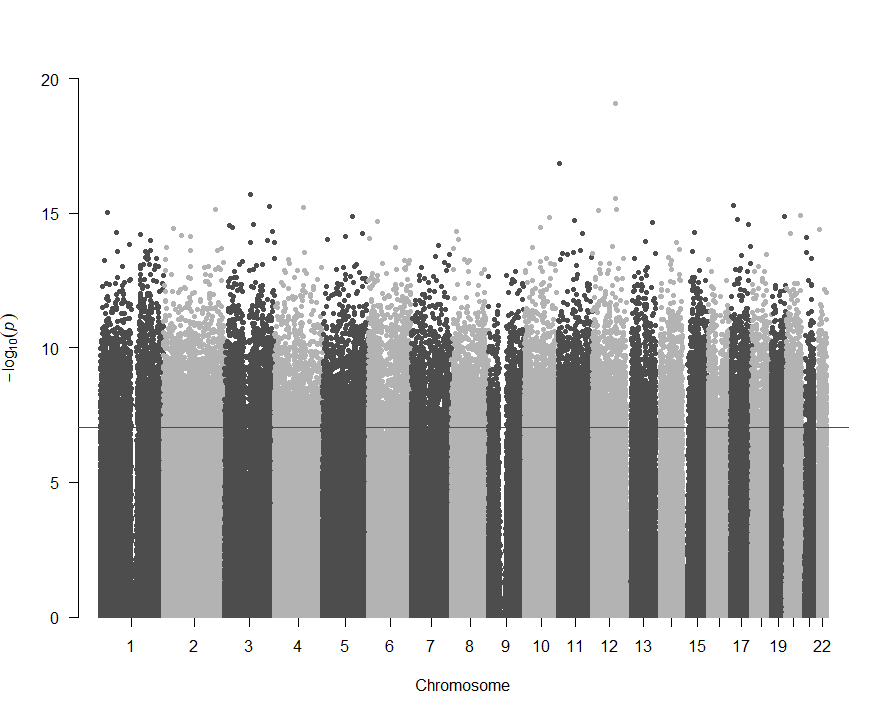


cg23812312

cg16500279 – *TRIM34*

cg03816901 – *LOC152225*

**SF7.** *Analysis 3* Manhattan Plot

**
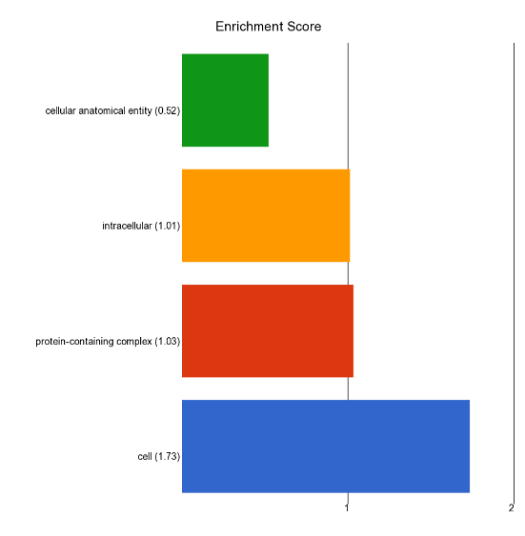

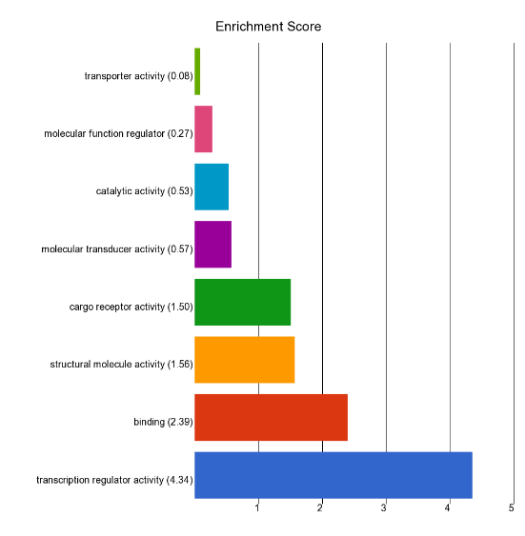
**

**
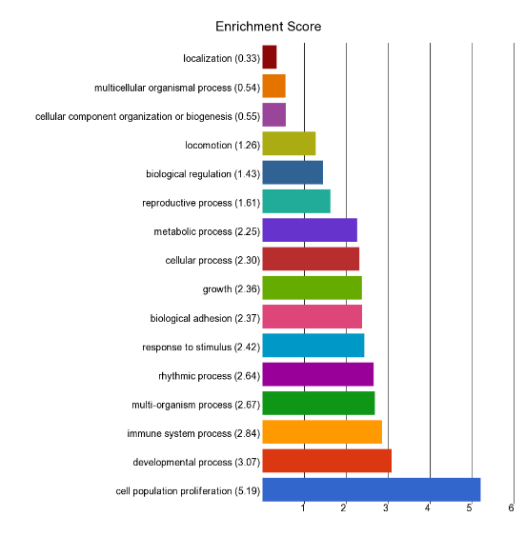
**

**SF 8a. GO analysis of biological processes SF 8b. GO analysis of cellular components SF 8c. GO analysis of molecular functions**

**SF8.** GO enrichment results for *Analysis 3*. Enrichment of top-ranked CpG sites for matched individuals with T1DM-ESKD (n=73) vs. T1DM (n=73): (FDR p-value ≤x10^-8^ and FC ±2). These gene classes are over-represented in the disease phenotype compared to a control gene set.

Abbreviations: CpG, cytosine-phosphate-guanine; ESKD, end-stage kidney disease; FC, fold change; FDR, false discovery rate; GO, gene ontology; T1DM, type 1 diabetes mellitus


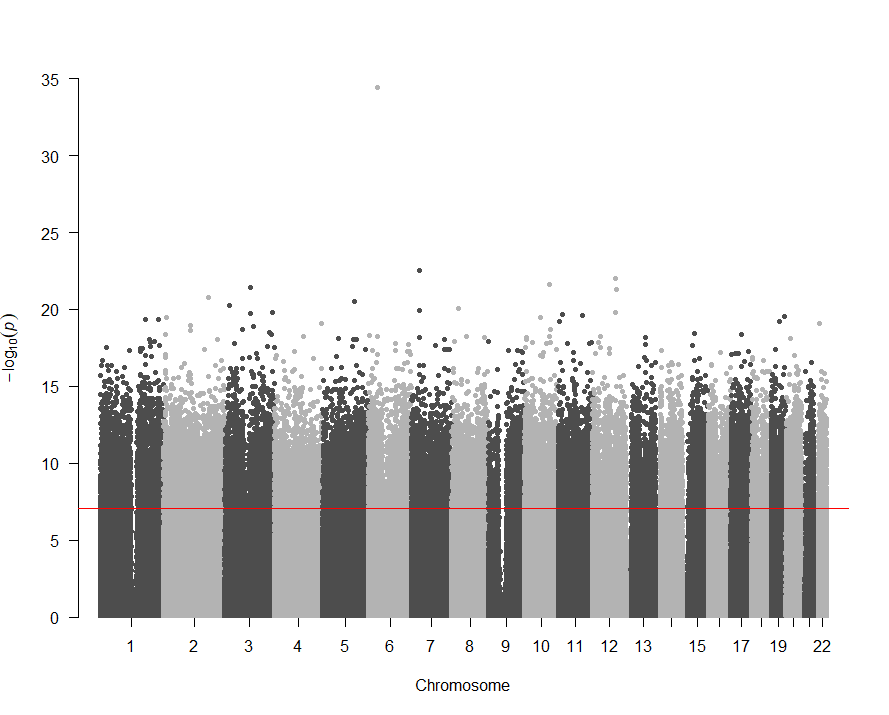


cg03546163 - *FKBP5*

cg23812312

cg11749142 - *MTURN*

**SF9.** *Analysis 4* Manhattan Plot


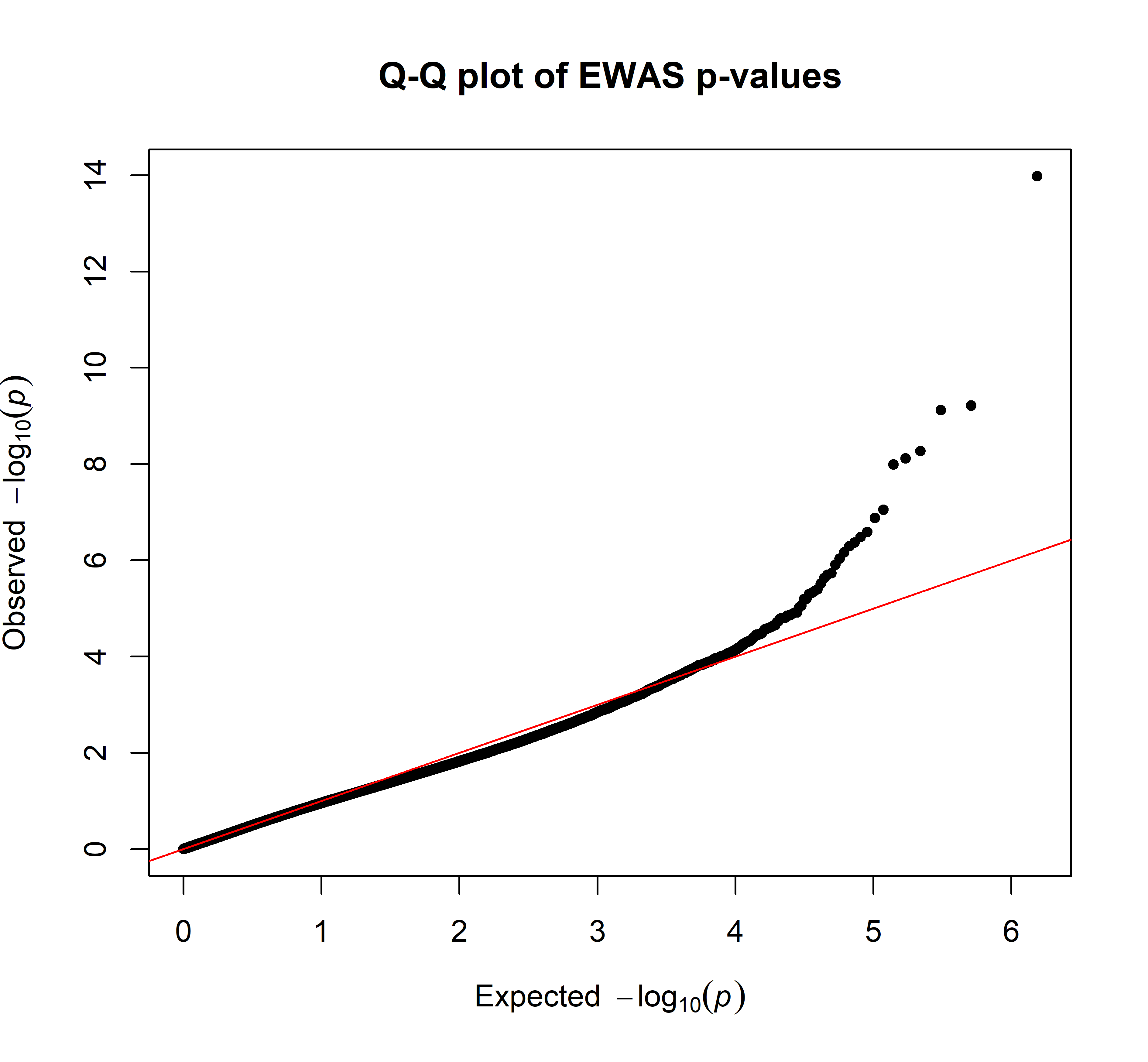


**SF10.** *Analyses 3 and 4* Q-Q Plot

**
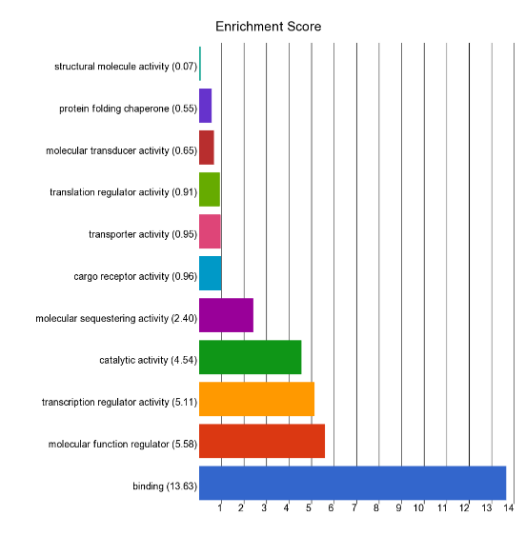

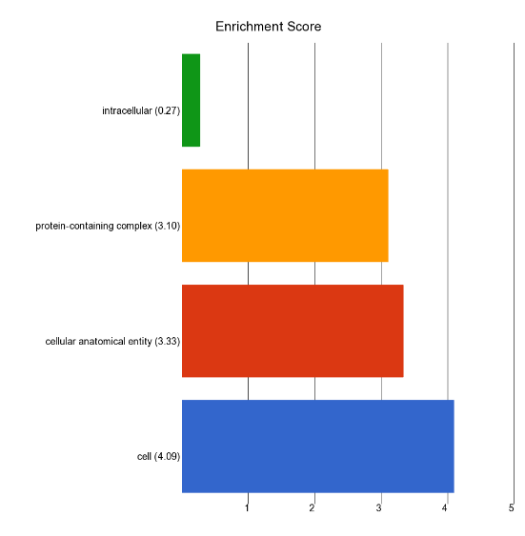

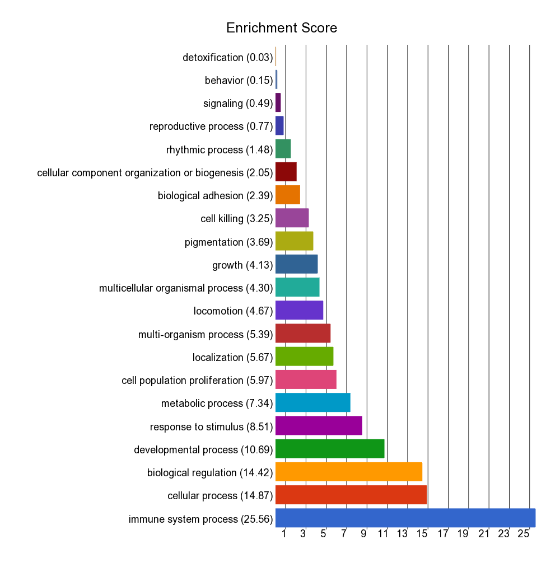
**

**SF 11a. GO analysis of biological processes SF 11b. GO analysis of cellular components SF 11c. GO analysis of molecular functions**

**SF11.** GO enrichment results for *Analysis 4*. Enrichment of top-ranked CpG sites for matched individuals with T1DM-ESKD (n=73) vs. T1DM (n=253): (FDR p-value ≤x10^-8^ and FC ±2). These gene classes are over-represented in the disease phenotype compared to a control gene set.

Abbreviations: CpG, cytosine-phosphate-guanine; ESKD, end-stage kidney disease; FC, fold change; FDR, false discovery rate; GO, gene ontology; T1DM, type 1 diabetes mellitus


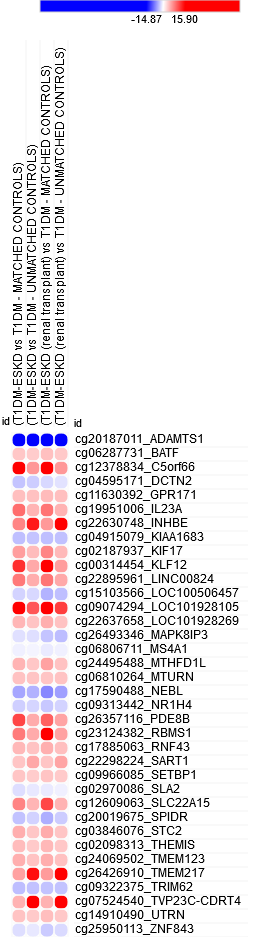


Hypo-

methylated

Hyper-

methylated

**SF12.** Heatmap of discovery dmCpGs in blood samples derived from T1DM ESKD versus T1DM individuals. Depicted dmCpGs are significant (p<0.05) in *Analyses* *1-4.*

Red = Hypermethylated; Blue = Hypomethylated.

Abbreviations: dmCpGs, differentially methylated cytosine-phosphate-guanine sites; ESKD, end-stage kidney disease;; T1DM, type 1 diabetes mellitus


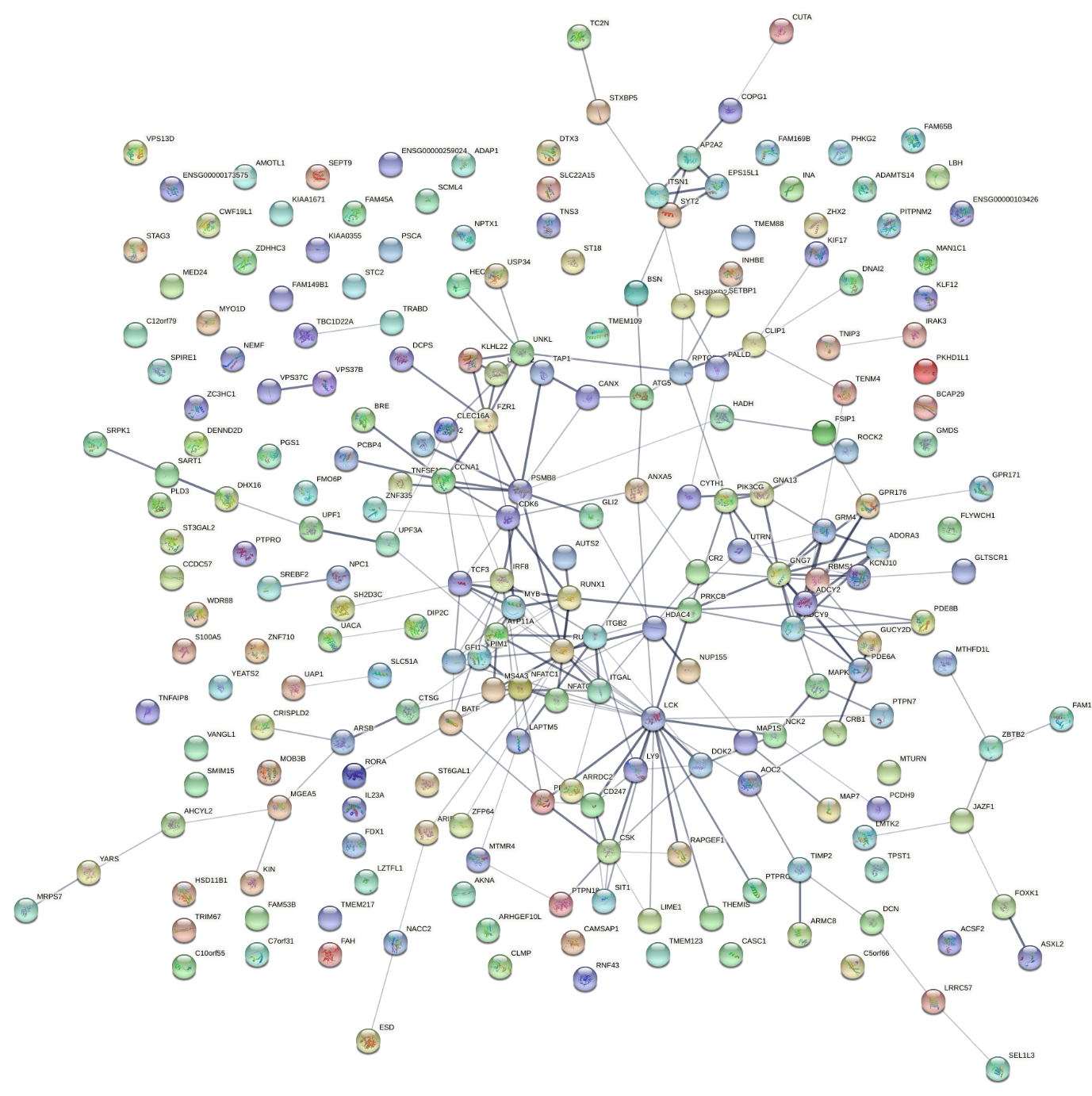


**SF13.** STRING analysis of genes which showed an increase in FC in individuals with T1D-ESKD from *Analysis 1*: matched individuals with T1DM-ESKD (n=107) vs. T1DM (n=107): FDR p≤x10^-8^ and FC±2 (ST4)

Abbreviations: ESKD, end-stage kidney disease; FC, fold change; FDR, false discovery rate; ST, Supplementary Table; T1DM, type 1 diabetes mellitus


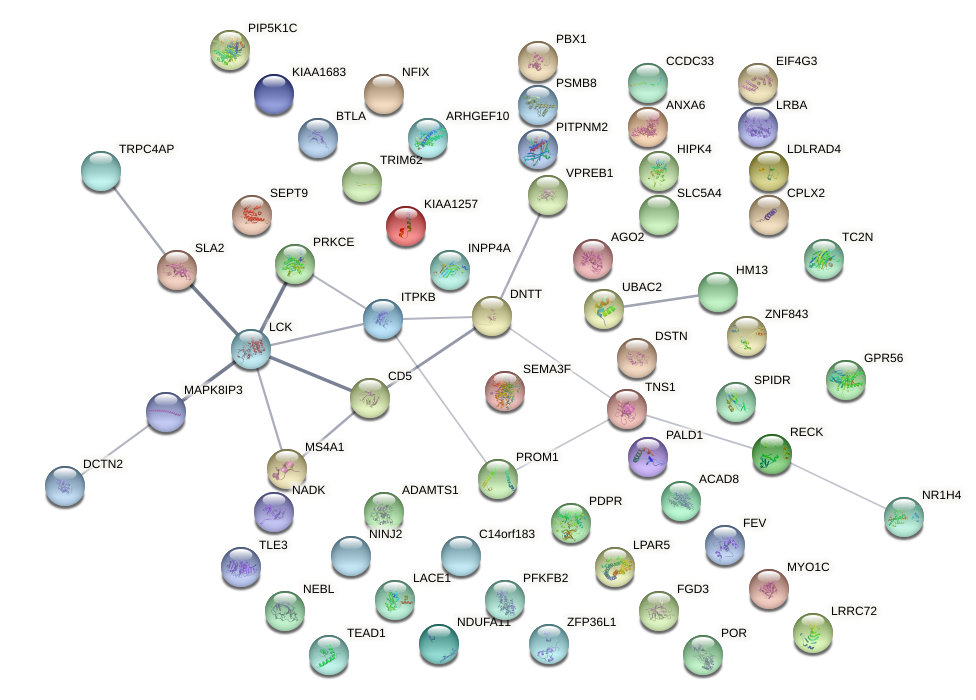


**SF14.** STRING analysis of genes which showed a decrease in FC in individuals with T1D-ESKD from *Analysis 1*: matched individuals with T1DM-ESKD (n=107) vs. T1DM (n=107): FDR p≤x10^-8^ and FC±2 (ST4)

Abbreviations: ESKD, end-stage kidney disease; FC, fold change; FDR, false discovery rate; ST, Supplementary Table; T1DM, type 1 diabetes mellitus


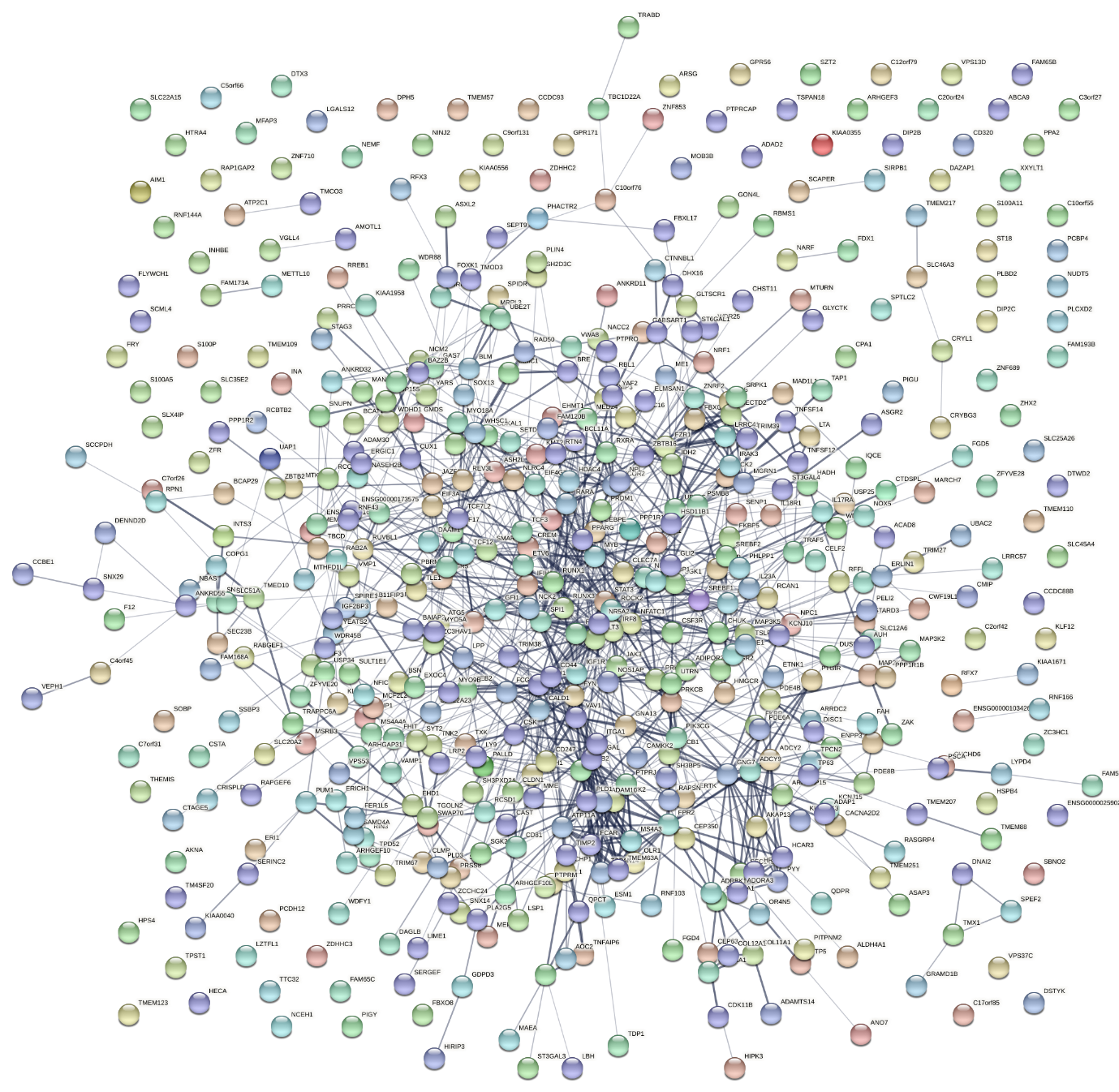


**SF15.** STRING analysis of genes which showed an increase in FC in individuals with T1D-ESKD from *Analysis 2*: individuals with T1DM-ESKD (n=107) vs. T1DM (n=253): FDR p≤x10^-8^ and FC±2 (ST12)

Abbreviations: ESKD, end-stage kidney disease; FC, fold change; FDR, false discovery rate; ST, Supplementary Table; T1DM, type 1 diabetes mellitus


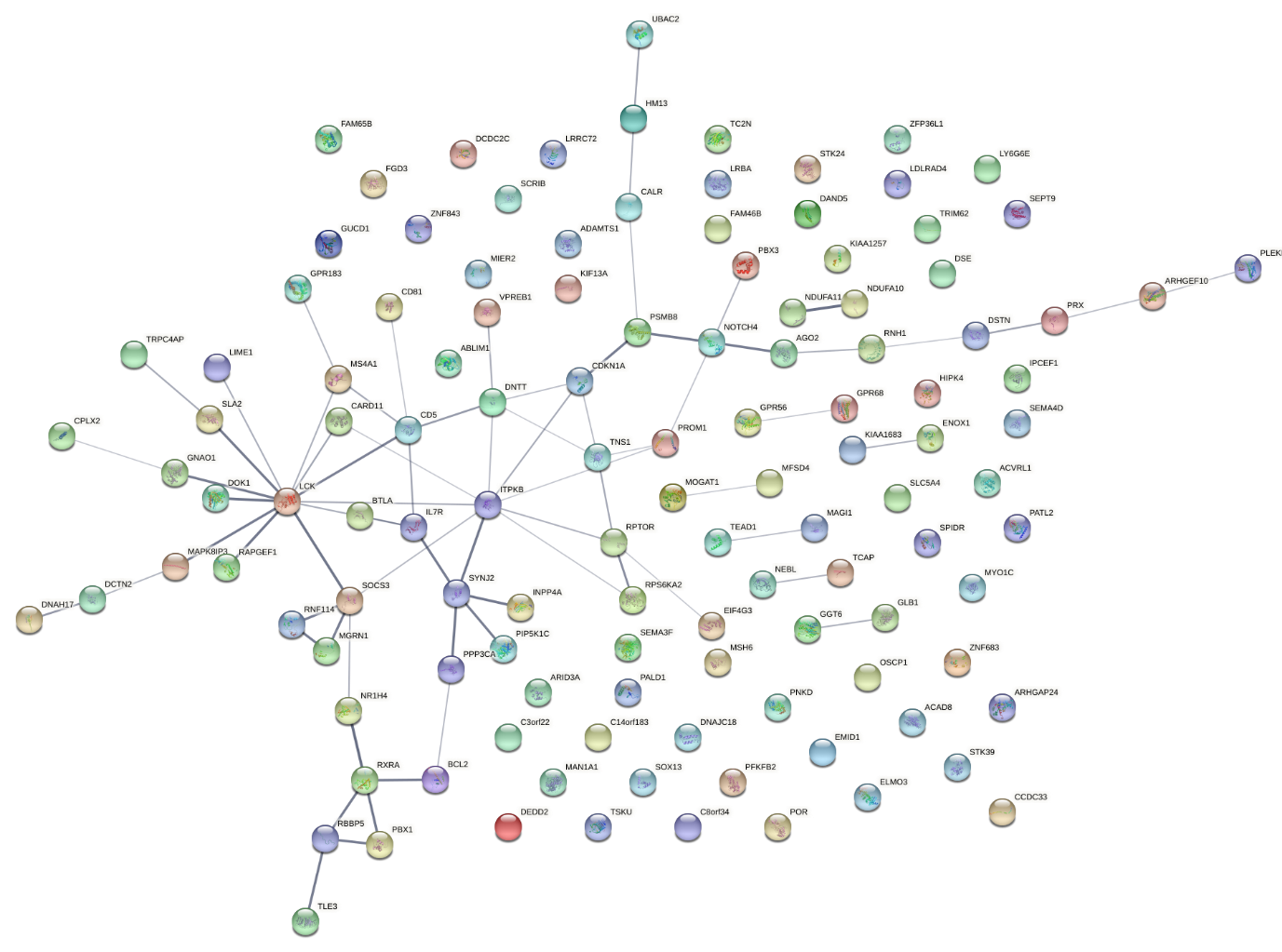


**SF16.** STRING analysis of genes which showed a decrease in FC in individuals with T1D-ESKD from *Analysis 2*: individuals with T1DM-ESKD (n=107) vs. T1DM (n=253): FDR p≤x10^-8^ and FC±2 (ST12)

Abbreviations: ESKD, end-stage kidney disease; FC, fold change; FDR, false discovery rate; ST, Supplementary Table; T1DM, type 1 diabetes mellitus


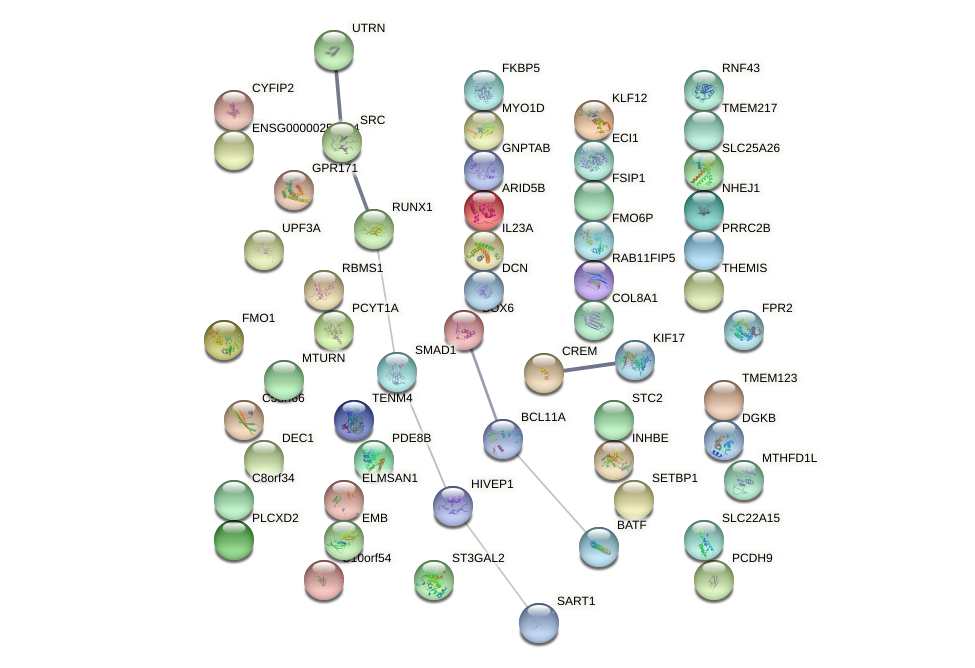


**SF17.** STRING analysis of genes which showed an increase in FC in individuals with T1D-ESKD from *Analysis 3*: matched individuals with T1DM-ESKD (n=73) vs. T1DM (n=73): FDR p≤x10^-8^ and FC±2 (ST21)

Abbreviations: ESKD, end-stage kidney disease; FC, fold change; FDR, false discovery rate; ST, Supplementary Table; T1DM, type 1 diabetes mellitus


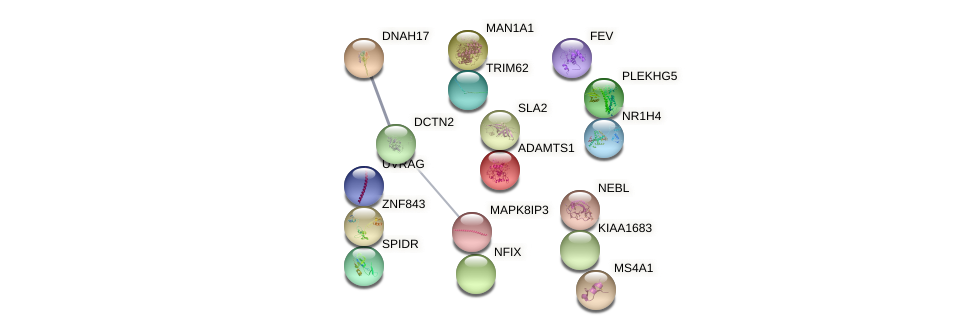


**SF18.** STRING analysis of genes which showed a decrease in FC in individuals with T1D-ESKD from *Analysis 3*: matched individuals with T1DM-ESKD (n=73) vs. T1DM (n=73): FDR p≤x10^-8^ and FC±2 (ST21)

Abbreviations: ESKD, end-stage kidney disease; FC, fold change; FDR, false discovery rate; ST, Supplementary Table; T1DM, type 1 diabetes mellitus


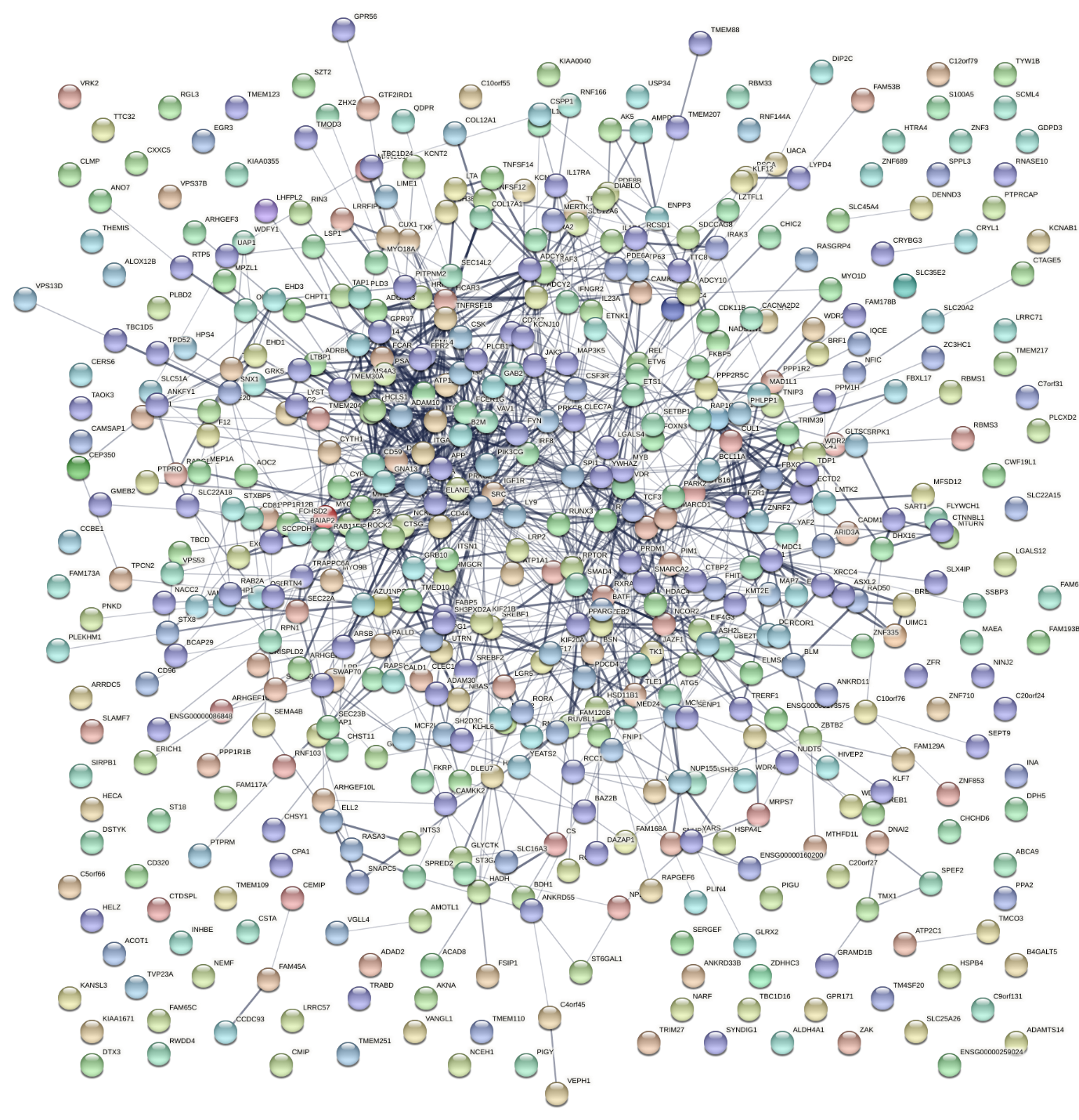


**SF19.** STRING analysis of genes which showed an increase in FC in individuals with T1D-ESKD from *Analysis 4*: individuals with T1DM-ESKD (n=73) vs. T1DM (n=253): FDR p≤x10^-8^ and FC±2 (ST29)

Abbreviations: ESKD, end-stage kidney disease; FC, fold change; FDR, false discovery rate; ST, Supplementary Table; T1DM, type 1 diabetes mellitus


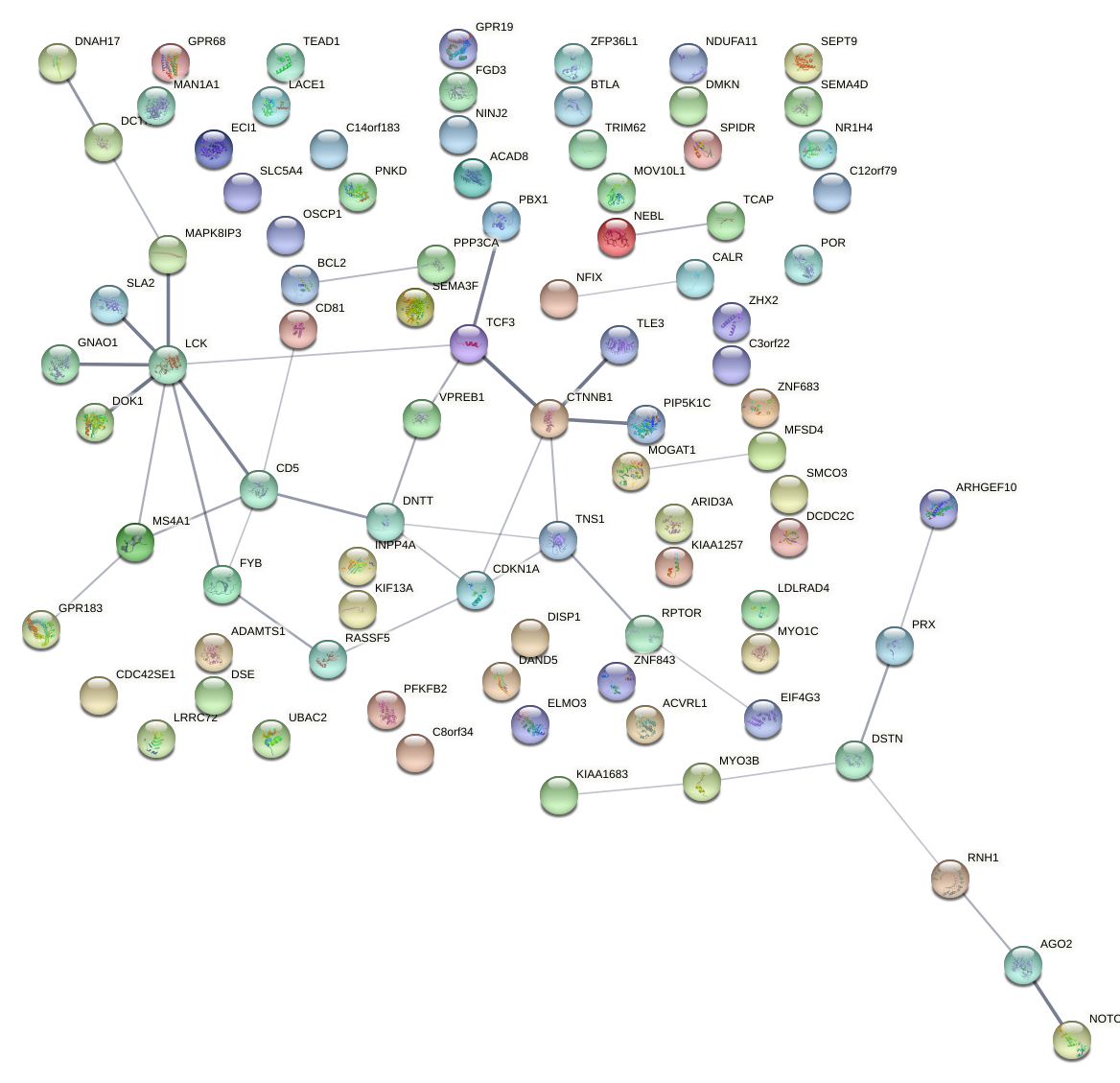


**SF20.** STRING analysis of genes which showed a decrease in FC in individuals with T1D-ESKD from *Analysis 4*: individuals with T1DM-ESKD (n=73) vs. T1DM (n=253): FDR p≤x10^-8^ and FC±2 (ST29)

Abbreviations: ESKD, end-stage kidney disease; FC, fold change; FDR, false discovery rate; ST, Supplementary Table; T1DM, type 1 diabetes mellitus


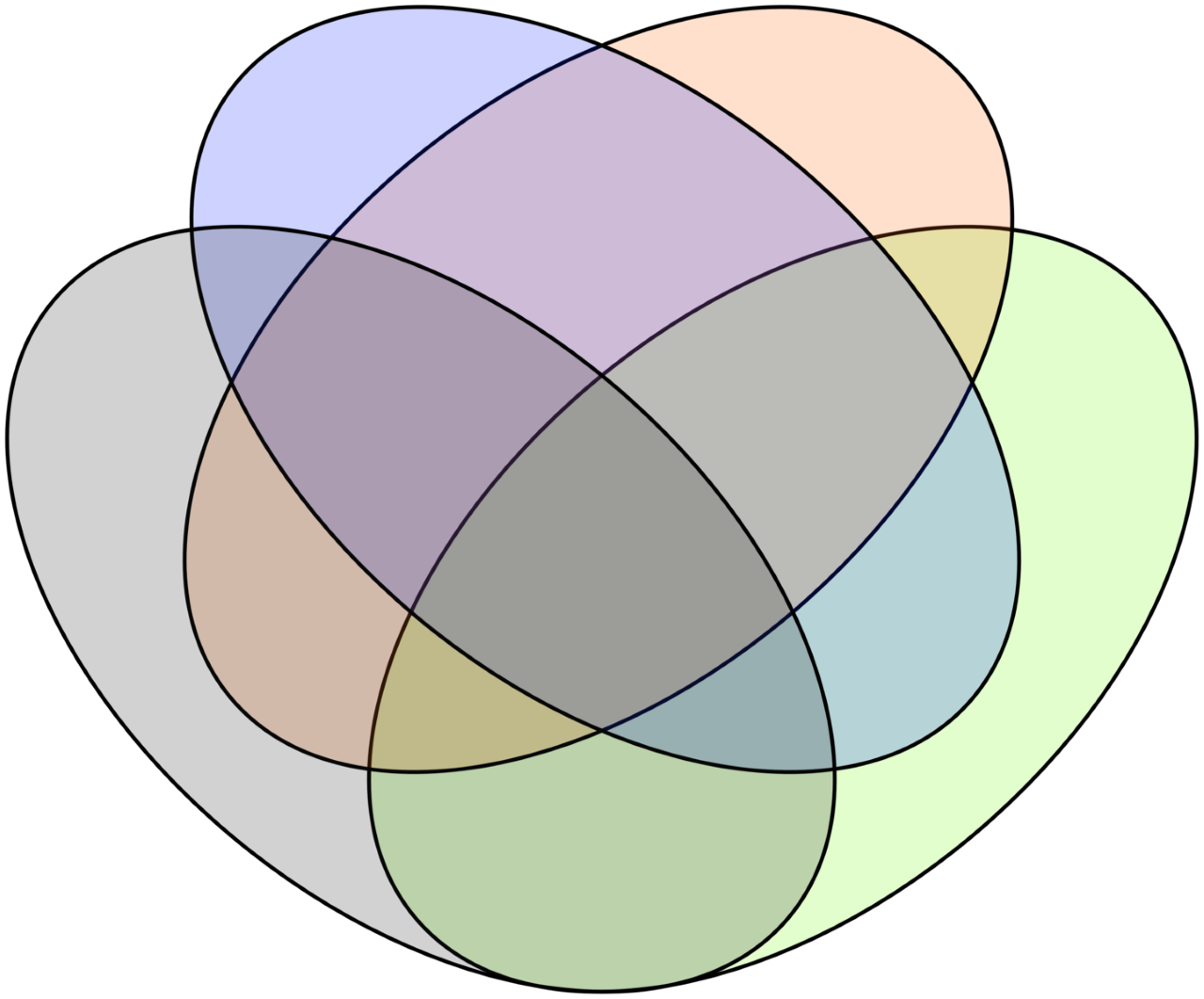
 **Analysis 2** **Analysis 3**

*PRKAG2 ^a^*

*PTPRN2 ^a,b,c,f^*

**Analysis 1** *ELMO1* ^a,c^ **Analysis 4**

*TAMM41*

*ARID5B*

*FKBP5 ^b,g^*

*RUNX3* ^a,b,c,g^ *CUX1 ^a,f^*

*UPF3A ^b^*

*AFF3, HDAC4 ^a^,*

*ITGAL ^b,c^, LY9 ^b,c^,*

*PBX1, PIM1 ^a^,*

*SEPTIN9 ^a,b,c,d,e,g^*

**SF21.** Venn diagram highlighting the genes in which top-ranked dmCpGs were located in individuals with T1DM-ESKD vs. T1DM (FDR p≤x10^-8^)

Footnote (functional support): a, eQTL support (p<x10^-5^) in Pima Indians (T2DM); b, glomerular kidney tissue support (p<0.05); c, tubular kidney tissue support (p<0.05); d, kidney tubule support for GFR slope (p<0.05) using 450K data; e, kidney tubule support for fibrosis (p<0.05) using 450K data; f, blood support for GFR (p<0.05) using 450K data; g, blood support for GFR slope (p<0.05) using 450K data.

Abbreviations: dmCpGs, differentially methylated CpG sites; eGFR, estimated glomerular filtration rate; eQTL, expression quantitative trait loci; ESKD, end-stage kidney disease; FDR, false discovery rate; T1DM, type 1 diabetes mellitus; T2DM, type 2 diabetes mellitus.


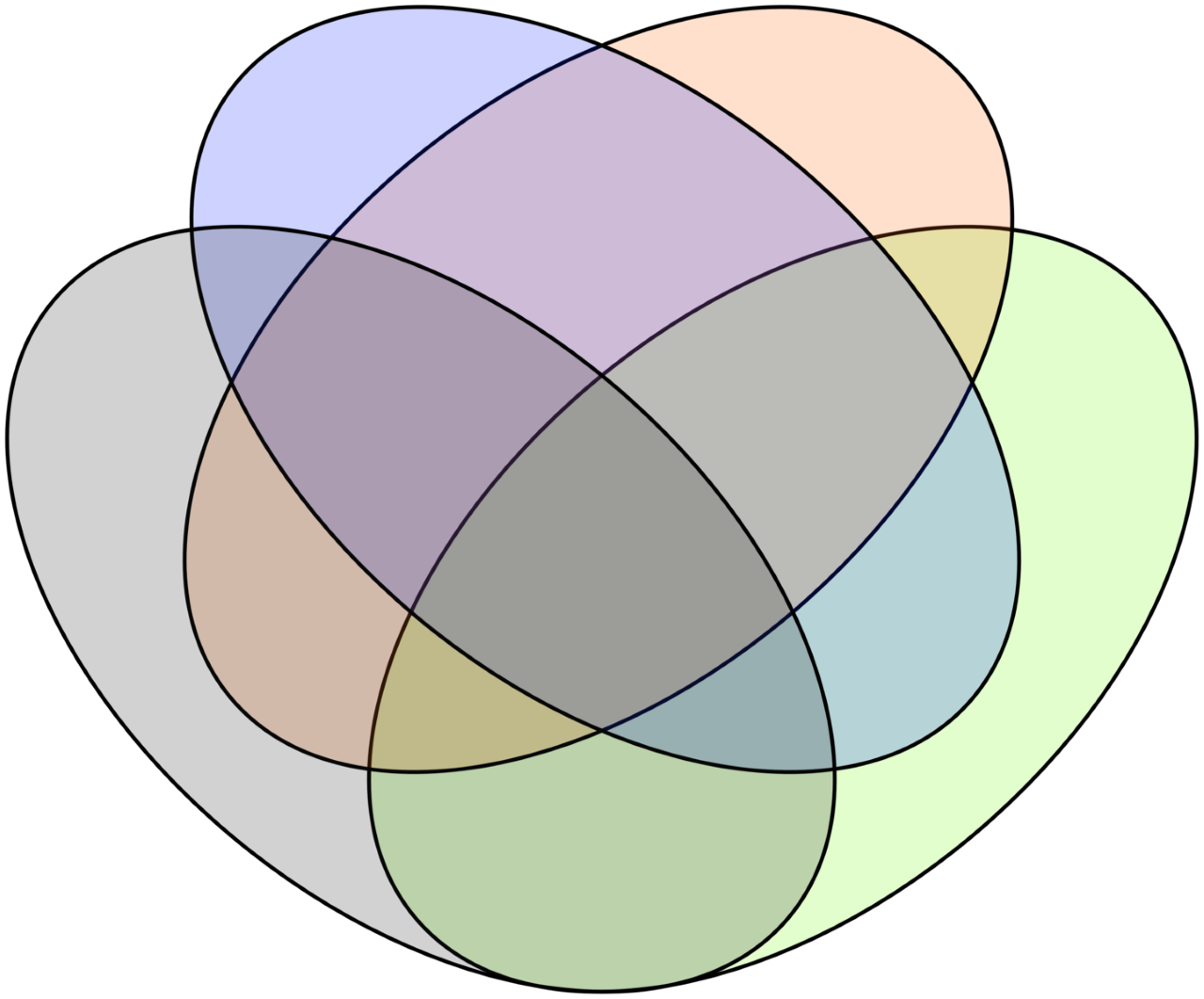
 **Analysis 2** **Analysis 3**

**Analysis 1** **Analysis 4**

*FKBP5 ^b,g^*

*ARID5B* *CUX1 ^a,f^*

*UPF3A ^b^*

*HDAC4 ^a^, ITGAL, ^b,c^*

*LY9 ^b,c^, PBX1,*

*PIM1 ^a^, RUNX3 ^a,b,c,g^,*

*SEPTIN9 ^a,b,c,d,e,g^*

**SF22.** Venn diagram highlighting the genes in which top-ranked dmCpGs were located in individuals with T1DM-ESKD vs. T1DM (FDR p≤x10^-8^ and FC ±2)

Footnote (functional support): a, eQTL support (p<x10^-5^) in Pima Indians (T2DM); b, glomerular kidney tissue support (p<0.05); c, tubular kidney tissue support (p<0.05); d, kidney tubule support for GFR slope (p<0.05) using 450K data; e, kidney tubule support for fibrosis (p<0.05) using 450K data; f, blood support for GFR (p<0.05) using 450K data; g, blood support for GFR slope (p<0.05) using 450K data.

Abbreviations: dmCpGs, differentially methylated CpG sites; eGFR, estimated glomerular filtration rate; eQTL, expression quantitative trait loci; ESKD, end-stage kidney disease; FC, fold change; FDR, false discovery rate; T1DM, type 1 diabetes mellitus; T2DM, type 2 diabetes mellitus.
